# 非小细胞肺癌免疫治疗疗效的预测生物标志物研究进展

**DOI:** 10.3779/j.issn.1009-3419.2024.106.12

**Published:** 2024-06-20

**Authors:** Tiansheng SUN, Zhang CHEN, Kunchen WEI, Hao TANG

**Affiliations:** 200003 上海，海军军医大学第二附属医院呼吸与危重症医学科; Department of Respiratory and Critical Care Medicine, Second Affiliated Hospital of Naval Medical University, Shanghai 200003, China

**Keywords:** 肺肿瘤, 免疫治疗, 肿瘤突变负荷, 生物标志物, 预测, Lung neoplasms, Immunotherapy, Tumor mutational burden, Biomarker, Prognostication

## Abstract

肺癌是世界上最常见的恶性肿瘤之一，其中非小细胞肺癌（non-small cell lung cancer, NSCLC）占大多数。免疫检查点抑制剂（immune checkpoint inhibitors, ICIs）的出现极大地改变了NSCLC的治疗策略，并改善了患者预后，然而现实中只有少部分患者能够获得长期受益。因此，确定可靠的预测生物标志物对选择治疗方式至关重要。随着近年来分子生物学、基因组测序技术的发展以及对肿瘤及其宿主免疫微环境的认识不断深入，生物标志物的研究层出不穷。本文围绕NSCLC免疫治疗疗效的预测生物标志物进行综述，以期为精准免疫治疗提供指导。

## Abstract (en)

Lung cancer is one of the most common malignant tumors in the world, of which non-small cell lung cancer (NSCLC) is the majority. The emergence of immune checkpoint inhibitors (ICIs) has greatly changed the treatment strategy of NSCLC and improved the prognosis of patients. However, in reality, only a small number of patients can achieve long-term benefit. Therefore, the identification of reliable predictive biomarkers is essential for the selection of treatment modalities. With the development of molecular biology and genome sequencing technology in recent years, as well as the in-depth understanding of tumor and its host immune microenvironment, research on biomarkers has emerged in an endless stream. This review focuses on the predictive biomarkers of immunotherapy efficacy in NSCLC, in order to provide some guidance for precision immunotherapy.

肺癌是全球范围内肿瘤相关死亡的主要原因^[1]^，在中国恶性肿瘤中发病率排名第一，其中非小细胞肺癌（non-small cell lung cancer, NSCLC）占80%-85%。按组织学分类，NSCLC最常见的亚型是腺癌，约占40%，而鳞状细胞癌占20%-30%^[2]^。众所周知，包括靶向程序性细胞死亡蛋白1（programmed cell death 1, PD-1）及其配体（programmed cell death ligand 1, PD-L1）和细胞毒性T淋巴细胞抗原-4（cytotoxic T lymphocyte-associated antigen-4, CTLA-4）为代表的免疫检查点抑制剂（immune checkpoint inhibitors, ICIs）已经彻底改变了晚期NSCLC的治疗策略，但60%-80%的NSCLC患者无法达到有效的缓解效果。如何通过生物标志物来指导个体化治疗以达到最大获益是免疫治疗领域面临的挑战，本文围绕NSCLC中ICIs疗效的相关预测生物标志物的研究进展进行综述。

## 1 肿瘤免疫原性相关生物标志物

### 1.1 PD-L1表达

肿瘤PD-L1表达是预测NSCLC免疫治疗疗效的关键标志物。III期KEYNOTE-024研究^[3]^表明，与含铂化疗相比，PD-L1表达≥50%且不伴有表皮生长因子受体（epidermal growth factor receptor, EGFR）突变或间变性淋巴瘤激酶（anaplastic lymphoma kinase, ALK）易位的晚期NSCLC患者，一线使用帕博利珠单抗能够获得较长的无进展生存期（progression-free survival, PFS）和总生存期（overall survival, OS）。KEYNOTE-042研究^[4]^将帕博利珠单抗免疫单药获益人群扩展到PD-L1表达≥1%的NSCLC患者。一项多中心回顾性研究^[5]^提示，在一线帕博利珠单抗单药治疗的NSCLC患者（PD-L1表达≥50%）中，PD-L1表达水平更高的患者有更好的疗效。同样一项荟萃分析^[6]^提示，与肿瘤细胞阳性比例分数（tumor cell proportion score, TPS）<1%、≥1%或1%-49%相比，TPS≥50%的患者从免疫治疗中获益更明显。尽管有以上发现，但PD-L1在预测免疫治疗疗效方面还是不够精准，比如在CheckMate 026^[7]^、MYSTIC^[8]^等研究中，研究者并未发现应用免疫治疗的PD-L1阳性患者的生存率高于化疗患者。分析原因可能是PD-L1既可以在肿瘤细胞中表达，也可以在免疫细胞中表达，并且存在瘤内和时空异质性，同时检测结果也易受主观解释因素及不同检测平台缺乏标准化的影响^[9]^。有研究^[10]^证实血浆可溶性PD-L1（soluble PD-L1, sPD-L1）可作为ICIs治疗的生物标志物，高sPD-L1对OS有负面影响，并且NSCLC较黑色素瘤、肾细胞癌或食管癌相关性更强，但也有研究^[11]^发现OS与治疗前sPD-L1水平无相关性。sPD-L1表达检测具有无创、安全的优点，然而目前相关研究较少，与免疫治疗疗效的相关性还需进一步研究证实。

### 1.2 肿瘤突变负荷（tumor mutational burden, TMB）

TMB是指特定区域内体细胞非同义突变的总数，高TMB与位于肿瘤细胞表面的人类白细胞抗原（human leukocyte antigen, HLA）分子新抗原的增加相关，进而增加了被新抗原反应T细胞识别的机会，因此ICIs可能在具有较高TMB的肿瘤中有更好的反应。CheckMate 227研究^[12]^显示，无论PD-L1表达水平如何，高TMB（≥10 mut/Mb）患者一线免疫联合治疗可延长PFS，论证了TMB可以作为NSCLC免疫治疗反应的潜在预测生物标志物，然而对KEYNOTE-021研究中队列C和G、KEYNOTE-189及KEYNOTE-407研究的探索性分析^[13]^显示TMB与免疫治疗疗效不相关。同样在一项回顾性队列研究^[14]^中，当TMB≥16 mut/Mb时，TMB值与OS、PFS、临床获益之间存在很强的正相关。然而，当TMB<16 mut/Mb时，增加的TMB值并未提高OS和PFS率。用于检测TMB的标本包括肿瘤组织和外周血。相比活检组织，血液TMB（blood TMB, bTMB）的优势为标本易获取并且便于动态监测。对两项大型随机试验（POPLAR和OAK）的回顾性分析研究^[15]^首次证明TMB可以在血浆中准确、可重复测量，并且bTMB与ICIs的临床获益相关，bTMB≥16 mut/Mb是NSCLC中具有临床意义的阈值，bTMB与高PD-L1表达无关，并且能够独立预测PFS获益。II期B-F1RST试验^[16]^中，bTMB≥16 mut/Mb组与<16 mut/Mb组之间PFS没有统计学差异，而bTMB≥16 mut/Mb有较高的客观缓解率（objective response rate, ORR）和更长的OS。TMB的检测方法包括全基因组测序、全外显子组测序、靶面板测序，但尚未在临床研究中标准化^[17]^，不同检测平台和检测基因数会影响检测结果。另外不同瘤种的免疫原性变化较大，针对所有瘤种的单一TMB阈值是否能够准确预测免疫治疗疗效一直受到争议。

### 1.3 微卫星不稳定（microsatellite instability, MSI）

微卫星是指存在于整个基因组中的短串联重复序列。MSI是错配修复缺陷（deficient mismatch repair, dMMR）导致的DNA错误复制中微卫星重复数目的改变。MSI严重程度可分为三类：微卫星稳定型（microsatellite stability, MSS）、低MSI型（MSI-low, MSI-L）和高MSI型（MSI-high, MSI-H）。MSI和dMMR具有较高的肿瘤免疫原性，dMMR状态的多种类型肿瘤对ICIs敏感^[18]^，这也促使帕博利珠单抗能够被批准用于存在MSI-H或dMMR状态的晚期实体瘤患者的治疗。然而其作为NSCLC免疫治疗疗效的生物标志物运用于临床，还需要针对NSCLC样本设计更多的前瞻性试验来验证。

## 2 肿瘤浸润淋巴细胞（tumor infiltrating lymphocytes, TILs）

肿瘤微环境（tumor microenvironment, TME）是一个动态变化的复杂系统，包括肿瘤细胞、炎症细胞、血管和细胞外基质。TILs是浸润肿瘤组织的所有淋巴细胞群，主要是指α、β T细胞（CD4^+^和CD8^+^），根据其位置可分为肿瘤内和肿瘤基质中的淋巴细胞^[19]^。TILs优先位于肿瘤细胞巢周围的基质区域，CD8^+ ^T细胞是丰度最高的亚群，较高密度的基质CD8^+^细胞毒性T细胞与较长的生存期相关，这种影响在PD-L1阳性病例中更为突出^[20]^。Zhang等^[21]^发现，CD8^+^ TILs、CD4^+^ TILs和干扰素-γ阳性的辅助型T细胞1（interferon-gamma^+ ^T helper 1 cells, IFN-γ^+^ Th1）是PFS和OS的阳性预测因子，而叉头框蛋白P3阳性的调节性T细胞（forkhead box P3^+^ regulatory T cells, Foxp3^+^ Treg）是显著的阴性预测因子。TILs在预测ICIs效能方面具有潜在的价值，然而评估TILs的标准方法是基于半定量评分方法的常规苏木素-伊红染色切片，该方法主观性强、重复性差。Rakaee等^[22]^开发了一种基于机器学习的TILs评分方法用于评估TILs与晚期NSCLC患者预后的相关性，显示高TILs水平与ICIs反应相关。未来人工智能和医学生物信息学研究的发展可能为评估TILs和TME功能发挥更大作用。

## 3 液体活检相关生物标志物

### 3.1 循环肿瘤DNA（circulating tumor DNA, ctDNA）

液体活检在肿瘤分子分析领域的应用越来越广泛。ctDNA是指血液中来源于肿瘤的游离DNA，可用于ICIs反应的早期评估。研究^[23]^发现ctDNA变异等位基因频率（variant allele frequencies, VAF）的变化与NSCLC的治疗时间以及临床结果密切相关，治疗早期ctDNA VAF的减少可能是预测免疫治疗长期获益的有效指标。Ricciuti等^[24]^对接受帕博利珠单抗±化疗的NSCLC患者进行了ctDNA评估，发现ctDNA变化与肿瘤靶病灶变化显著相关，VAF的减少与更高的ORR、更长的PFS和OS相关。Yang等^[25]^发现免疫治疗前血浆中未检测到ctDNA的NSCLC患者有更长的OS。Moding等^[26]^研究发现，在化疗后ctDNA可测的患者中接受巩固免疫者比未接受巩固免疫治疗的预后更好，治疗早期ctDNA水平降低的患者预后优于ctDNA水平升高患者，治疗后ctDNA不可测的患者的PFS更长。此外，ctDNA也可以作为一种无创手段来检测与免疫治疗敏感性相关的基因点突变。ctDNA可通过多种技术进行分离和分析，目前主要方法是以聚合酶链式反应或高通量测序为核心技术，限制ctDNA临床应用的主要问题是缺乏统一的风险分层阈值，以及多种检测方法的覆盖深度、片段大小等各不相同^[9]^。

### 3.2 循环肿瘤细胞（circulating tumor cells, CTCs）

CTCs是从原发肿瘤脱落后渗入血液并在血液中循环的肿瘤细胞，可以单细胞或以成簇的方式迁移，并且可提供有关肿瘤的生物学信息。治疗期间CTCs计数的变化可以反映NSCLC治疗的敏感性，有研究^[27]^发现在基线和治疗4周时存在CTCs是预后较差的独立预测因素。与ctDNA相比，CTCs具有完整的细胞形态和细胞内物质保存更完整的优点，可以提供转录组学、基因组学和蛋白质组学信息，因此对CTCs的分析可更加多元，结果的置信度也更高^[9]^，从而为肿瘤预后预测提供更多的信息。CTCs核心技术主要包括捕获和富集、检测和识别以及释放，其中较为关键的是捕获和富集，可以分为基于物理特性和基于生物学特性的方法，前者成本相对较低，但效率低下、纯度差且缺乏特异性，而后者能获取单个CTC形态学、细胞学和遗传学表征，但易受上皮标志物表达水平的影响导致富集率降低而不准确。随着微流控芯片、纳米材料和下一代测序技术的发展，CTCs相关技术也取得较快的进步^[28]^，高通量测序和单细胞RNA测序等研究也有助于CTCs组学研究在未来应用到NSCLC的免疫治疗中。

### 3.3 外泌体（exosomes）

细胞外囊泡是一组亚微米膜结合的细胞器，存在于血液以及脑脊液、羊水、尿液等体液中，其最常见的亚型是外泌体，直径为40-160 nm，来源于正常细胞或肿瘤细胞，在增强增殖、上皮-间充质转化、转移、血管生成以及影响TME和抑制免疫应答等方面发挥作用^[29]^。一项纳入黑色素瘤（n=18）和NSCLC（n=8）患者的研究^[30]^发现治疗2个月后，完全缓解（complete response, CR）和部分缓解（partial response, PR）组血浆来源外泌体PD-L1信使RNA（messenger RNA, mRNA）水平较基线下降（P=0.016），疾病稳定（stable disease, SD）组改变无统计学意义（P=0.586），而在疾病进展（progressive disease, PD）组较基线升高（P=0.001）。一项纳入30例抗PD-1/PD-L1治疗的EGFR/ALK野生型晚期NSCLC患者的研究^[31]^发现，与健康人群相比，NSCLC患者的外泌体微小RNA（microRNA, miRNA）表达谱有明显改变，并且PD组较PR组治疗前基线水平的hsa-miR-320d、hsa-miR-320c和hsa-miR-320b明显较高。总之，外泌体的PD-L1状态是预测NSCLC患者免疫治疗的一种有前景的生物标志物。外泌体的检测技术主要有动态光散射、纳米粒子跟踪分析、透射电子显微镜、蛋白质免疫印记、高通量测序等，然而其分离和纯化方法的标准化问题限制了其临床实践。

## 4 新兴ICIs治疗靶点的相关生物标志物

### 4.1 淋巴细胞活化基因-3（lymphocyte activation gene-3, LAG-3）

LAG-3是在免疫细胞上表达的一种细胞表面分子，负调控T细胞增殖和效应T细胞功能。LAG-3和PD-1是不同的抑制性免疫检查点，在RELATIVITY-047试验^[32]^中，Relatlimab（抗LAG-3抗体）-Nivolumab联合或Nivolumab单药治疗黑色素瘤患者，LAG-3的表达被量化为肿瘤区域（包括肿瘤、间质和侵袭边缘）中阳性染色的免疫细胞与肿瘤区域中所有有核细胞的比例，且样本中至少含有100个活的肿瘤细胞。结果发现在两个治疗组中，LAG-3表达≥1%的患者中位PFS估计更长，而无论LAG-3表达如何，Relatlimab-Nivolumab组表现优于Nivolumab组。然而，另一项II期研究^[33]^表明肿瘤样本基线LAG-3表达并不能预测LAG-3/PD-1抗体的疗效。LAG-3表达能否作为抗LAG-3/PD-1治疗特异性的预测性生物标志物需要进一步的研究确认。

### 4.2 T细胞免疫球蛋白和黏蛋白结构域3（T-cell immunoglobulin and mucin domain-3, TIM-3）

TIM-3是一种共抑制性细胞表面受体，主要表达在产生IFN-γ的T细胞质膜上，也可以表达在包括Treg细胞、巨噬细胞、树突状细胞以及白血病干细胞等免疫细胞上。当TIM-3与配体结合时，免疫细胞或适应性免疫细胞的成熟和活化减弱，有利于肿瘤细胞的增殖和存活^[34]^。Sabatolimab（抗TIM-3抗体）在晚期实体瘤中单独和联合使用抗PD-1抗体的I/IB期临床试验^[35]^检测了CD8、PD-L1、CD163、LAG-3和TIM-3的表达以及与TIM-3通路相关的RNA测序、基因特征、T细胞或IFN-γ特征，然而并未观察到这些潜在的免疫生物标志物与治疗反应之间的关系。值得注意的是，3例有反应的患者活检样本TIM-3表达>10%。然而，由于相关的研究较少，并且样本量和对治疗有反应的患者数量少，很难对这些生物标志物进行可靠的评估。

### 4.3 T细胞免疫球蛋白与ITIM结构域（T cell immunoglobulin and ITIM domain, TIGIT）

TIGIT是一种具有抑制功能的新型免疫检查点受体，在T细胞和自然杀伤细胞上表达。在不同的细胞类型和细胞定位中，TIGIT表达存在着高度差异，简单评估肿瘤样本中的TIGIT表达可能无法确定其在肿瘤免疫微环境中的相关性。很少有研究以定量和空间分辨方式评估TIGIT表达，多数研究采用了非客观且可重复性差的方法，例如常规免疫组化，而没有进一步研究TIGIT表达和/或其配体在TME表型的相关性^[36]^。目前，TIGIT定量表达与抗TIGIT治疗反应的相关性值得进一步研究。

## 5 影像组学

影像组学通过提取影像的定量特征，挖掘肿瘤的生物学信息，可用于预测肿瘤分型及预后。在一项研究^[37]^中，研究者从患者治疗前计算机断层扫描（computed tomography, CT）中对肿瘤进行视觉分析和影像组学特征提取，发现使用临床变量和影像组学特征组合构建的模型预测PD-L1表达的能力高于仅使用临床变量的预测模型。影像组学研究不仅对预测PD-L1的表达状态有着良好的表现，在预测ICIs疗效方面也有一定价值。朱振宸等^[38]^提取抗PD-1/PD-L1治疗的NSCLC患者治疗前动脉期CT图像多病灶影像组学，通过基于注意力机制的多示例学习算法获得加权组学特征，采用Logistic回归建立加权评分模型，该模型在预测治疗疗效方面高于PD-L1-1模型、PD-L1-50模型以及临床模型。Peng等^[39]^在2个中心264例ICIs治疗的NSCLC患者影像中提取感兴趣的肿瘤区域的三个子区域放射学特征，构建次区域放射组学模型（sub-regional radiomics model, SRRM），发现SRRM较常规放射组学、PD-L1表达和TMB评分有更好的预测性能，且SRRM能有效地对患者的PFS风险进行分层。

影像组学中人工智能的发展开阔了发现生物标志物的视野，初步凸显了其应用前景，但缺乏高质量证据的研究支持，还需要设计从开发、验证到整合到临床实践全过程的前瞻性研究进行论证^[40]^。

## 6 其他生物标志物

### 6.1 基因突变

携带V-raf鼠肉瘤病毒癌基因同源体B（v-raf murine sarcoma viral oncegene homolog B, BRAF）突变或Kirsten大鼠肉瘤病毒癌基因同源物（Kirsten rats arcomaviral oncogene homolog, KRAS）和肿瘤蛋白P53（tumor protein 53, TP53）共突变的NSCLC患者从ICIs中获益最多，而EGFR或ALK突变或c-ros肉瘤致癌因子-受体酪氨酸激酶（ROS proto-oncogene 1, receptor tyrosine kinase, ROS1）重排通常与较低的PD-L1水平和TMB、TME的免疫浸润以及对ICIs耐药有关^[41]^。一项回顾性研究^[42]^中，ICIs单药治疗组丝/苏氨酸激酶11（serine/threonine kinase 11, STK11）和Janus激酶（recombinant janus kinase, JAK）2突变与早期进展的更高可能性相关，ICIs联合化疗组细胞周期蛋白依赖性激酶抑制因子2A（cyclin dependent kinase inhibitor 2A, CDKN2A）突变与较差的长期预后相关。同样，有研究^[43]^发现27.8%的免疫治疗病例发现了包括STK11、β-2-微球蛋白（β-2-microglobulin, B2M）、大肠癌结肠癌聚阳性基因（adenomatous polyposis coli, APC）、哺乳动物雷帕霉素靶蛋白（mammalian target of rapamycin, mTOR）、Kelch样ECH关联蛋白1（Kelch-like ECH-associated protein 1, KEAP1）和JAK1/2的耐药突变。然而，需要进一步的研究来论证这些驱动基因突变可作为ICIs疗效的潜在预测生物标志物。

### 6.2 肠道菌群

肠道微生物群的组成可能会影响肿瘤免疫反应以及对免疫治疗的疗效。Routy等^[44]^报道，肠道微生物群中嗜黏蛋白阿克曼菌种的丰度与免疫治疗反应存在正相关，抗生素的使用则会抑制免疫治疗的临床效益。其他研究也证实了肠道微生物群作为ICIs生物标志物的作用，Zhao等^[45]^收集了抗PD-1联合化疗的NSCLC患者和健康人的粪便样本，结果发现NSCLC患者和健康人在肠道微生物的β多样性和代谢途径上有显著差异，有临床获益的患者中，双歧杆菌、大肠杆菌和沙门氏菌明显富集。与基线时未检测出双歧杆菌breve组相比，检出组的PFS明显更长。有研究^[46]^结果显示，肠道微生物群的代谢物丁酸盐可促进抗PD-1治疗的疗效，是一个非常有前景的生物标志物。肠道微生物群动力学在预测各种恶性肿瘤抗PD-1/PD-L1治疗反应方面的潜在作用，为未来的大型前瞻性研究提供了思路。虽然通过支气管肺泡灌洗液、唾液或粪便样本分析患者的微生物组尚未常规应用于临床实践，但仍鼓励在研究条件下使用^[47]^。

### 6.3 T细胞受体（T cell receptor, TCR）

TCR中存在的不同互补决定区域的巨大多样性是由变量（V）、多样性（D）和连接（J）基因的体细胞重组产生的，这可能与对ICIs的反应有关，目前已经开发了TCR的高通量测序来评估肿瘤患者TCR库的多样性^[48]^。一项对抗PD-1/PD-L1治疗的NSCLC患者细胞中分离的TCRβ链的互补决定区3进行测序，发现ICIs治疗前PD-1^+^CD8^+^TCR多样性高的患者对ICIs治疗反应更好，并有更长的PFS，治疗后PD-1^+^CD8^+^TCR克隆性增加的患者PFS更长^[49]^。然而，另一项研究^[50]^发现在EGFR/ALK野生型NSCLC患者亚群中，与无应答者（SD或PD）相比，应答者（PR）治疗后TCR多样性明显下降，TCR多样性减少的患者显示出更长的PFS。外周血中TCR库的分析和监测可能作为筛选ICIs治疗受益患者的替代标志物，但仍需要进一步探索和验证。

### 6.4 外周血细胞

对于接受免疫治疗的NSCLC患者，基线时低绝对中性粒细胞计数、高绝对嗜酸性粒细胞计数与更好的预后有关^[51]^，相对嗜酸性粒细胞计数（relative eosinophil count, REC）增加的患者疾病控制率显著高于REC未增加的患者^[52]^。外周血细胞比值对免疫治疗也有一定的预测价值，如低中性粒细胞/淋巴细胞比值（neutrophil to lymphocyte ratio, NLR）或衍生的NLR（derived NLR, dNLR）与生存结局改善相关，而高血小板/淋巴细胞比值（platelet to lymphocyte ratio, PLR）、低淋巴细胞/单核细胞比值（lymphocyte to monocyte ratio, LMR）与更短的OS相关^[51]^。外周血细胞计数的优点是无创且易获取，但易受感染等因素影响较大，并且相应的阈值仍需要进一步大样本研究证实。

## 7 总结与展望

虽然PD-L1仍是当前免疫治疗首选的生物标志物，但PD-L1表达在预测免疫治疗方面仍表现欠佳，寻找经济性强、预测性能良好的生物标志物显然是NSCLC免疫治疗领域的现实考验。生物标志物样本主要有活检组织来源的有创标本和以液体样本为主的无创样本，组织样本检测相对准确性高，但来源易受限制，且不容易监测随访；而液体样本获取相对容易并且可动态观测，但存在着肿瘤细胞难以富集、检测方法未标准化等问题。一些新兴的生物标志物初步展示潜力，但缺乏相对大样本的前瞻性研究，目前结论可靠性较差。

鉴于肿瘤免疫微环境的复杂多样性以及当前研究的局限，单一生物标志物往往缺乏敏感性和特异性。多变量的结合，例如肺免疫预后指数（lung immune prognostic index，LIPI；根据dNLR和乳酸脱氢酶水平评估）、改良后格拉斯哥预后分数（modified Glasgow prognostic score，mGPS；根据血清C反应蛋白和白蛋白评估）等评分预测模型，证实有较好的预测价值。另外多组学、多参数结合能进一步提高预测效能，已有不少研究尝试将影像学、病理学和基因组学等多模态整合分析。随着对肿瘤免疫微环境的研究更加深入，新的免疫治疗靶点不断发现，以及生物医学技术不断进步，生物标志物采集、检测等技术难题不断被破解，相信在不久的未来，更加精准、有效的生物标志物能被发现，进而指导临床医生制定治疗方案，使更多的NSCLC患者从免疫治疗中获益。
